# Regulation of Stomatal Tropism and Infection by Light in *Cercospora zeae-maydis*: Evidence for Coordinated Host/Pathogen Responses to Photoperiod?

**DOI:** 10.1371/journal.ppat.1002113

**Published:** 2011-07-28

**Authors:** Hun Kim, John B. Ridenour, Larry D. Dunkle, Burton H. Bluhm

**Affiliations:** 1 Department of Plant Pathology, University of Arkansas, Fayetteville, Arkansas, United States of America; 2 Crop Production and Pest Control Research Unit, USDA-ARS, Purdue University, West Lafayette, Indiana, United States of America; Michigan State University, United States of America

## Abstract

*Cercospora zeae-maydis* causes gray leaf spot of maize, which has become one of the most widespread and destructive diseases of maize in the world. *C. zeae-maydis* infects leaves through stomata, which is predicated on the ability of the pathogen to perceive stomata and reorient growth accordingly. In this study, the discovery that light was required for *C. zeae-maydis* to perceive stomata and infect leaves led to the identification of *CRP1*, a gene encoding a putative blue-light photoreceptor homologous to White Collar-1 (WC-1) of *Neurospora crassa*. Disrupting *CRP1* via homologous recombination revealed roles in multiple aspects of pathogenesis, including tropism of hyphae to stomata, the formation of appressoria, conidiation, and the biosynthesis of cercosporin. *CRP1* was also required for photoreactivation after lethal doses of UV exposure. Intriguingly, putative orthologs of *CRP1* are central regulators of circadian clocks in other filamentous fungi, raising the possibility that *C. zeae-maydis* uses light as a key environmental input to coordinate pathogenesis with maize photoperiodic responses. This study identified a novel molecular mechanism underlying stomatal tropism in a foliar fungal pathogen, provides specific insight into how light regulates pathogenesis in *C. zeae-maydis*, and establishes a genetic framework for the molecular dissection of infection via stomata and the integration of host and pathogen responses to photoperiod.

## Author Summary

Fungal diseases of crop plants are a significant threat to global food security. Improving host resistance is the most cost-effective and environmentally sound strategy for sustainable disease management. However, many devastating diseases of important crops have proven impossible to manage through genetic resistance alone, thus underscoring the need for new strategies to improve resistance. For example, although many fungal pathogens utilize natural openings in leaves to infect host plants, little is known about the underlying molecular dialogue between the host and the pathogen, or whether resistance during this stage of pathogenesis can be improved through breeding. In this study, we explored the ability of a common fungal foliar pathogen of maize to sense and penetrate pores in the leaf epidermis involved in gas exchange (stomata). We determined that light is required for the fungus to sense stomata and identified a blue-light photoreceptor in the fungus that mediates stomatal tropism and infection. Intriguingly, orthologous photoreceptors in other fungi entrain endogenous circadian rhythms, and plants are known to regulate stomatal aperture in response to daily cycles of light and darkness. This study raises the distinct possibility that plants and pathogens coordinate their responses to photoperiod, thus providing a novel insight into fungal pathogenesis.

## Introduction

Each year, foliar diseases caused by plant pathogenic fungi cause incalculable losses to global food production. Despite the significance of fungal foliar diseases, the current understanding of how fungi infect plants has important gaps. Arguably, the fungal foliar pathogen in which infection is most thoroughly investigated at the molecular level is *Magnaporthe oryzae*, which generates enormous turgor pressure inside specialized infection structures known as appressoria to directly penetrate the epidermis of rice leaves [1]. While informative, this model is only fractionally representative of how foliar fungal pathogens infect plants. Many fungi exclusively infect leaves through natural openings such as stomata [2], although the molecular basis of this phenomenon is not fully understood. As early as 1905, experiments conducted with artificial leaf surfaces in which drilled holes were substituted for stomata revealed that penetration of stomata by rust fungi is thigmotropic, *i.e.*, regulated by changes in leaf topography associated with stomata, a finding that was substantiated during ensuing decades of research [3]–[5]. However, mechanisms explaining how non-thigmotropic fungi such as *Cercospora* spp. find their way into stomata were debated for more than a half-century. In 1916, Pool and McKay postulated that hyphae of the sugar beet pathogen *C. beticola* sense nearby stomata, possibly through chemoattraction [6]. Their hypothesis resulted from observations that hyphae of *C. beticola* nearly always took the shortest possible path to gain entry to the closest stomata, and defied the conventional wisdom of the era, namely that hyphae of *C. beticola* and most other fungi encountered stomata randomly [7]. In the late 1970s and early 1980s, histological studies performed with *C. beticola* and *C. zeae-maydis* confirmed that reorientation of hyphal growth to stomata was non-thigmotropic [8], [9]. These results supported the original idea of stomatal tropism advanced by Pool and McKay, and today, debate over the existence of non-thigmotropic stomatal tropism in fungi has been largely put to rest [10].

Somewhat surprisingly, despite the importance of stomata as portals of entry, relatively little is known mechanistically about how pathogens find and penetrate stomata or how plants defend themselves against this method of attack. There is ample evidence that foliar pathogens other than fungi display stomatal tropism, including certain bacteria and oomycetes [11], [12]. For example, *Pseudomonas syringae* moves towards stomata during infection of *Arabidopsis*, which the bacterium utilizes to infiltrate mesophyll tissues of leaves. However, upon perceiving the pathogen, the plant closes its stomata as a component of the defense response [12]. To usurp this mechanism of plant defense, the bacterium produces coronatine, a secondary metabolite that induces calcium channel opening in guard cells, thus leading to an increase in stomatal aperture-which, in turn, allows the pathogen access to mesophyll tissues [12]. What is known of the interaction between *P. syringae* and *Arabidopsis* gives rise to fascinating questions regarding foliar fungal pathogens that infect leaves through stomata. For example, are the mechanisms through which fungi and bacteria sense stomata similar? Do fungal foliar pathogens produce secondary metabolites structurally or functionally analogous to coronatine that alter stomatal aperture? Are plants able to defend themselves against stomatal invasion by fungi as was recently demonstrated in bacteria?

Information about molecular mechanisms underlying infection of leaves by fungi through stomata may come from a more thorough understanding of the association between light and fungal pathogenesis. Although plants regulate stomatal aperture in response to environmental cues such as temperature and humidity, the basal regulation of stomatal aperture is circadian, governed by an endogenous molecular clock that is entrained by daily cycles of day and night [13]. Plants have evolved complex molecular mechanisms to perceive changes in the quality, quantity, and periodicity of light, and these signal transduction pathways also regulate stomatal aperture [14], [15]. Fungi have also developed sensitive molecular mechanisms to detect light. Most known fungal responses to light are mediated by blue (∼410–520 nm) light and are regulated by members of the *white collar-1* (*wc-1*) family of photoreceptor-encoding genes [16], [17]. Originally identified in *Neurospora crassa*, *wc-1* encodes a dual-function blue light photoreceptor/transcription factor that forms a heterodimer with the protein encoded by *white collar-2* to form the White Collar Complex (WCC). The WCC governs virtually all of the light-dependent responses in *N. crassa*, including the innate circadian clock [16], [17]. Genes similar to *wc-1* have subsequently been identified in numerous fungi, suggesting that the molecular mechanisms underlying responses to light may be at least partially conserved across the fungal kingdom, although few light-responsive genes with clear roles in foliar pathogenesis have been identified.

In this study, we investigated molecular mechanisms underlying pathogenesis in the maize foliar pathogen *Cercospora zeae-maydis*, with particular emphasis on understanding the molecular basis of infection through stomata. An initial discovery that light was required for stomatal tropism and infection in *C. zeae-maydis* led to the discovery of a putative blue-light photoreceptor (encoded by *CRP1*) homologous with WC-1 of *N. crassa*. Functional characterization of *CRP1* through targeted mutagenesis revealed that blue light regulates multiple aspects of pathogenesis, and that some, but not all, blue-light responses in *C. zeae-maydis* are regulated by *CRP1*. Our findings identify a novel molecular mechanism through which fungi utilize light as a signal to regulate stomatal tropism and pathogenesis, which has led to the formulation of new hypotheses regarding the coordination of fungal pathogenesis and plant defense responses during the initiation and development of foliar diseases.

## Results

### Light is required for stomatal tropism and foliar infection in *C. zeae-maydis*

**Figure 1:**
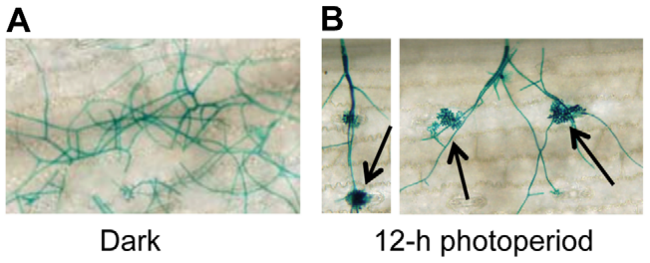
*C. zeae-maydis* requires light to form appressoria and infect maize. A GUS-labeled reporter strain of *C. zeae-maydis* was inoculated on maize leaves, and incubated for four days in either **A,** 12 h light/dark cycles, or **B,** constant darkness. Black arrows indicate appressoria.

To gain insight into environmental factors influencing stomatal tropism and infection through stomata, a series of experiments was conducted in which the influence of temperature, relative humidity, and photoperiod on pathogenesis were explored. Unexpectedly, plants exposed to constant darkness failed to develop visible signs of infection. To further dissect this observation, we utilized a GUS-labeled reporter strain of *C. zeae-maydis* to assess the effect of constant darkness on distinct stages of pathogenesis, including germination of conidia, formation of appressoria, and the initiation of necrotic lesions. In constant darkness, germination of conidia was not significantly different than in light/dark cycles (data not shown). However, in constant darkness, *C. zeae-maydis* failed to form appressoria (Figure 1A), whereas the fungus displayed stomatal tropism and formed appressoria normally when exposed to a 12 hr light/dark cycle (Figure 1B). From these observations, we concluded that light is required for infection, although further experimentation was required to conclusively determine whether light was specifically required for the induction of appressorium formation, or whether light was required for the fungus to sense stomata, in which case the defect in appressorium formation could have been an indirect result from incubation in darkness.

### Cercosporin biosynthesis and conidiation in *C. zeae-maydis* is regulated by blue light

**Figure 2:**
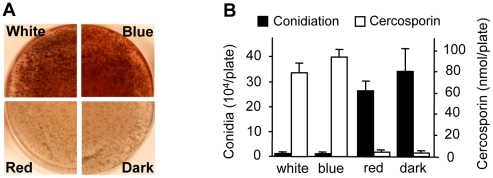
Effects of light on cercosporin biosynthesis and conidiation of *C. zeae-maydis*. **A,** Conidia (10^5^) were spread on 0.2× PDA medium, and the plates were incubated for four days in constant white light, blue light (400–530 nm), red light (600–700 nm), or darkness. Cercosporin accumulated in the medium as a dark red pigment. **B,** Conidiation and cercosporin were measured after four days in cultures grown on V8-agar and 0.2× PDA, respectively.

To gain additional clues into the molecular regulation of light responses in *C. zeae-maydis*, we evaluated the effect of specific wavelengths of light on morphogenesis and secondary metabolism. On favorable culture media, the formation of asexual spores (conidia) is repressed by constant light in *C. zeae-maydis*, whereas constant darkness induces conidiation [18]. Conversely, the biosynthesis of cercosporin, a photosensitizing perylenequinone that accumulates as a red pigment in culture, is known to be induced by light in *Cercospora* spp. but is produced in very low levels in constant darkness [19], [20]. To identify specific wavelength(s) of light responsible for these phenomena, we constructed customized fungal growth chambers with acrylic filters that specifically transmitted blue, green, orange, or red light. However, because the transmission spectrum of the green-light filters overlapped with the blue-light filters, and the transmission spectrum of the orange-light filters overlapped with the red-light filters, we focused on defining blue- and red-light responses to avoid potential ambiguities associated with spectral overlap. Cultures of *C. zeae-maydis* grown exclusively in either white or blue light synthesized high levels of cercosporin, whereas cercosporin biosynthesis in cultures grown exclusively in red light was indistinguishable from cultures grown in constant darkness (Figure 2A). Additionally, cultures grown exclusively in constant blue light consistently displayed an earlier onset of cercosporin biosynthesis and higher levels of biosynthesis compared to cultures grown in constant white light (data not shown). In contrast to cercosporin biosynthesis, cultures grown in constant white or blue light failed to produce conidia on conducive media, whereas cultures grown in constant red light or darkness produced abundant amounts of conidia (Figure 2B). To determine if responses to blue light were conserved among *Cercospora* species, we evaluated cercosporin biosynthesis and conidiation in *C. beticola*, *C. kikuchii*, and *C. sorghi*. Conidiation and cercosporin biosynthesis in these three species was similar to *C. zeae-maydis* in response to light (Figure S1, Table S1), indicating that the regulation of cercosporin biosynthesis and conidiation by blue light is at least partially conserved throughout the genus. Together, these findings demonstrated that blue light plays a key role in regulating at least two critical stages of pathogenesis among *Cercospora* spp. and thus implicated blue light-responsive signaling pathways in foliar pathogenesis.

### The genome of *C. zeae-maydis* contains a putative ortholog of the *N. crassa* gene *wc-1*

**Figure 3:**
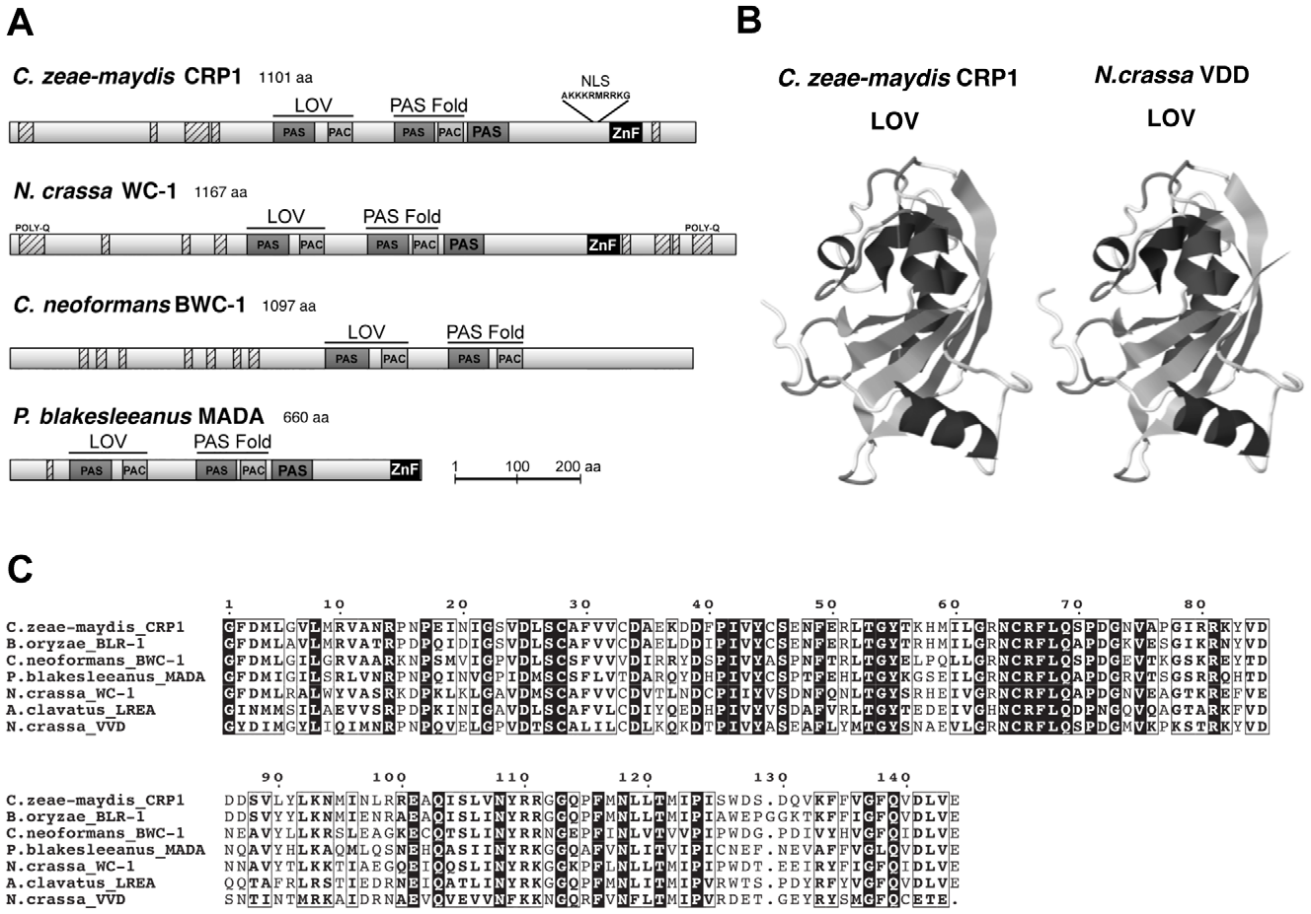
*CRP1* encodes a putative ortholog of the *N. crassa* protein WC-1. **A,** Conceptual translation of *CRP1* revealed a protein predicted to contain at least four functional domains (LOV, PAS-Fold, PAS, and ZF) represented by shaded boxes. Diagrams of WC-1 and putative orthologs are presented to illustrate homology. Boxes with hash marks represent predicted low-complexity regions, NLS indicates a nuclear localization signal. **B,** Comparison of LOV domains from Crp1 of *C. zeae-maydis* and VVD of *N. crassa*. **C,** Alignment of the LOV domains encoded by *vvd* and putative *wc-1* orthologs from fungi.

Observations that cercosporin biosynthesis and conidiation were regulated specifically by blue light focused gene discovery efforts on putative blue-light photoreceptors in *C. zeae-maydis*, with the ultimate goal of gaining a molecular foothold into understanding stomatal tropism and appressorium formation in response to light. In many filamentous fungi, the well-characterized white collar regulatory complex (WCC) originally characterized in *N. crassa* is essential for blue-light-mediated processes [21]–[23]. The WCC consists of the interacting transcription factors White Collar-1 (WC-1) and White Collar-2 (WC-2), of which WC-1 is the limiting component of the complex [24], [25]. To clone the *wc-1* ortholog from *C. zeae-maydis*, we performed PCR with degenerate primers designed from highly conserved regions of *wc-1* and putative orthologs identified in sequenced Dothidiomycete genomes. After cloning and sequencing a small segment of a putative ortholog in *C. zeae-maydis*, the remainder of the gene (designated *CRP1* for *Cercospora* regulator of pathogenesis) was obtained by genome-walker PCR and sequencing cosmid clones. Conceptual translations of *CRP1* indicated that the gene contains an open reading frame (ORF) of 3,535 bp. Additionally, comparison of the *CRP1* ORF with mRNA from *wc-1* and putative orthologs from other filamentous fungi indicates that the *CRP1* ORF contains four putative introns; intron 1 (50 bp), intron 2 (62 bp), intron 3 (45 bp), and intron 4 (72 bp) starting 1,245 bp, 1,732 bp, 2,831 bp, and 3,163 bp from the start site of the ORF, respectively. Conceptual translation of *CRP1* also revealed a predicted protein of 1,101 amino acid residues with a predicted molecular weight of 120.01 kDa and isoelectric point of pH 8.20 (Figure 3). Crp1 is predicted to contain a nuclear localization signal residing at amino acid residues 954–963 [26] (Figure 3A). Global comparison of the amino acid residues of Crp1 and WC-1 indicate the two proteins share 49.9% similarity and 37.0% identity. Additionally, the two proteins are predicted to share a similar domain architecture [27], [28] (Figure 3A). Further evidence supporting the function of Crp1 as a blue-light photoreceptor was obtained through comparisons of the conceptually translated protein with WC-1 and Vivid (VVD) of *N. crassa*. Crp1 is predicted to contain a Light, Oxygen, Voltage (LOV) domain, a Per-ARNT-Sim (PAS)-Fold domain, a PAS domain, and a Zinc-finger DNA binding domain. The flavin-binding LOV domain, a member of the PAS domain superfamily, has been implicated in sensing and responding to environmental stimuli [29], [30]. The LOV domain is well conserved in numerous organisms, including bacteria, fungi, and plants; exceptional conservation is found within the fungal kingdom [29], [31] (Figure 3B). VVD of *N. crassa* is a blue-light photoreceptor of 186 amino acids that contains a single LOV domain [32], [33]. The LOV domain of VVD has been characterized extensively [30], [32]. Ribbon diagrams depicting the tertiary structure of the LOV domain contained in VVD of *N. crassa* and the predicted tertiary structure of the LOV domain contained in Crp1 indicated a percent identity of 45.27% and an e value of 8.30e-36 [30], [34] (Figure 3C). Moreover, Crp1 contains two additional highly conserved domains involved in light signaling and protein interaction – a PAS-Fold domain and a PAS domain [24], [35], [36]. In addition to the three light sensing domains, Crp1 contains a conserved Zinc-finger DNA binding domain common among GATA transcription factors that regulate changes in gene expression in response to light [21], [37]. The most striking difference in functional regions between Crp1 and WC-1 is the apparent lack of the WC-1 terminal poly-glutamine (Poly-Q) regions in Crp1 (Figure 3A). In WC-1, the Poly-Q terminal regions are thought to function in transcriptional activation, as in the GATA family transcription factors [38]. The lack of the terminal Poly-Q regions in Crp1 could indicate a novel mechanism of activation in response to light. Intriguingly, Crp1 does contain a number of low-complexity regions (LCR) near its terminal regions (Figure 3A). The LCRs are implicated in numerous biologically important functions, including protein-protein interactions, transcription, transcriptional regulation, and translation [39].

**Figure 4:**
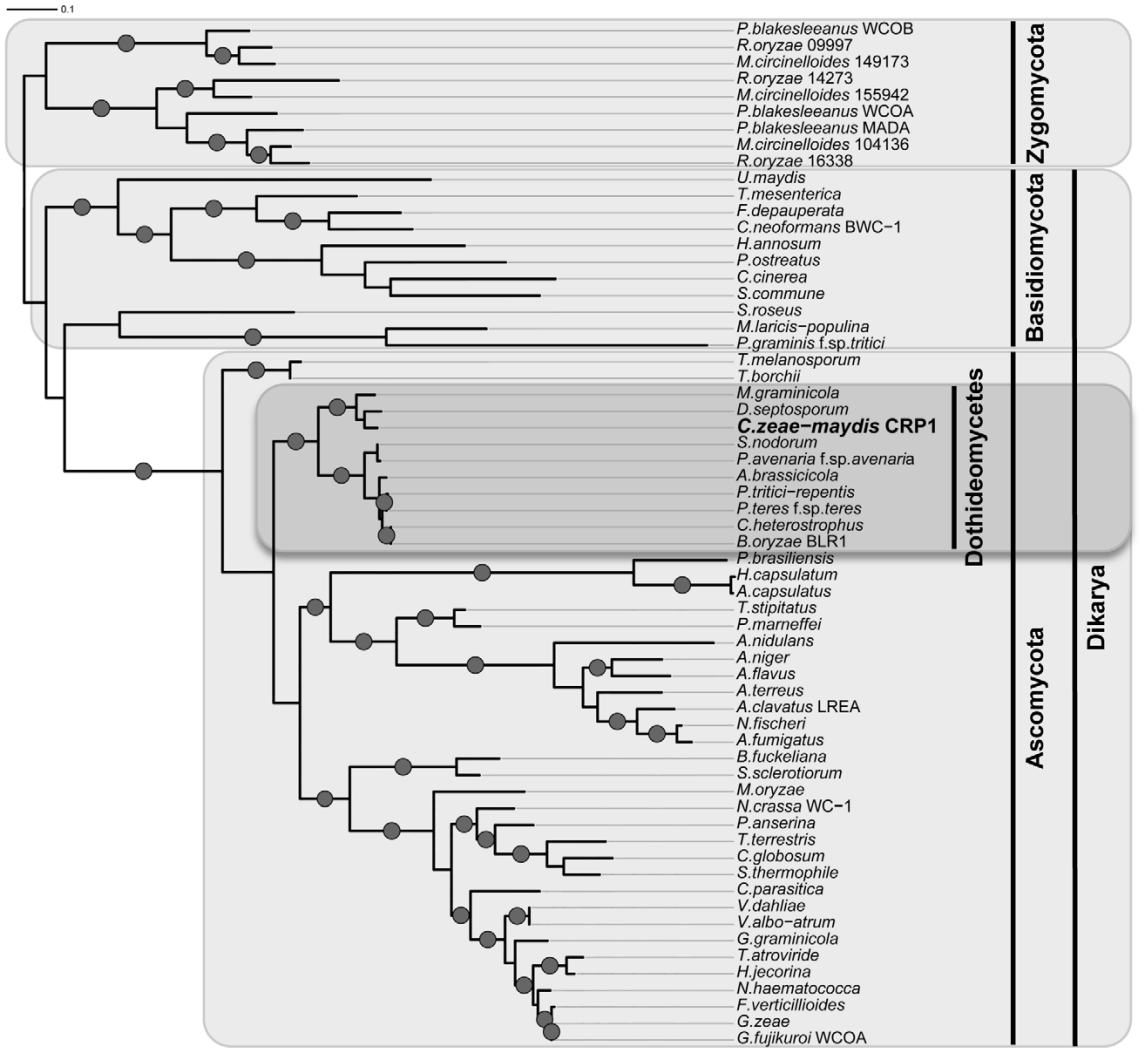
Phylogenetic analysis of Crp1-like proteins. Maximum likelihood tree inferred from Crp1-like proteins within the Zygomycota and the fungal subkingdom Dikarya. RAxML ML bootstrap values are represented by solid dots at each node where the value is above 80%.

BLAST analysis (tblastx) querying the translated sequence of *CRP1* against the GenBank nucleotide collection indicated that *CRP1* shares high sequence similarity with characterized and predicted genes in a wide range of fungi within the clades Ascomycota, Basidiomycota, and Zygomycota. The highest sequence similarity was obtained from species within the subdivision Pezizomycotina, and Crp1 groups tightly within a clade formed by Dothideomycetes [40] (Figure 4). Subsequent protein-protein BLAST (blastp) analyses of individual fungal genomes revealed that most fungi from within the Ascomycota and Basidiomycota have one putative ortholog of *CRP1*, although members of the Zygomycota examined in this study possessed three genes encoding proteins highly similar to Crp1. In addition, members of the Ascomycota subphyla Saccharomycotina and Taphrinomycotina [41], which include both the budding and fission yeasts, do not encode a protein orthologous to Crp1. Notably, most of the divergent regions of the putative orthologs were found near the N- and C-termini; the central region containing both the LOV domain and the PAS-Fold domain were highly conserved in all orthologous proteins examined (Figure 3A). A phylogenetic analysis of Crp1-like proteins within the Zygomycota and the fungal subkingdom Dikarya produced a well-supported tree corresponding closely to the branch order expected from fungal phylogeny [42], [43], indicating the analyzed sequences likely arose from a common ancestor (Figure 4).

### *CRP1* regulates stomatal tropism and formation of appressoria

**Figure 5:**
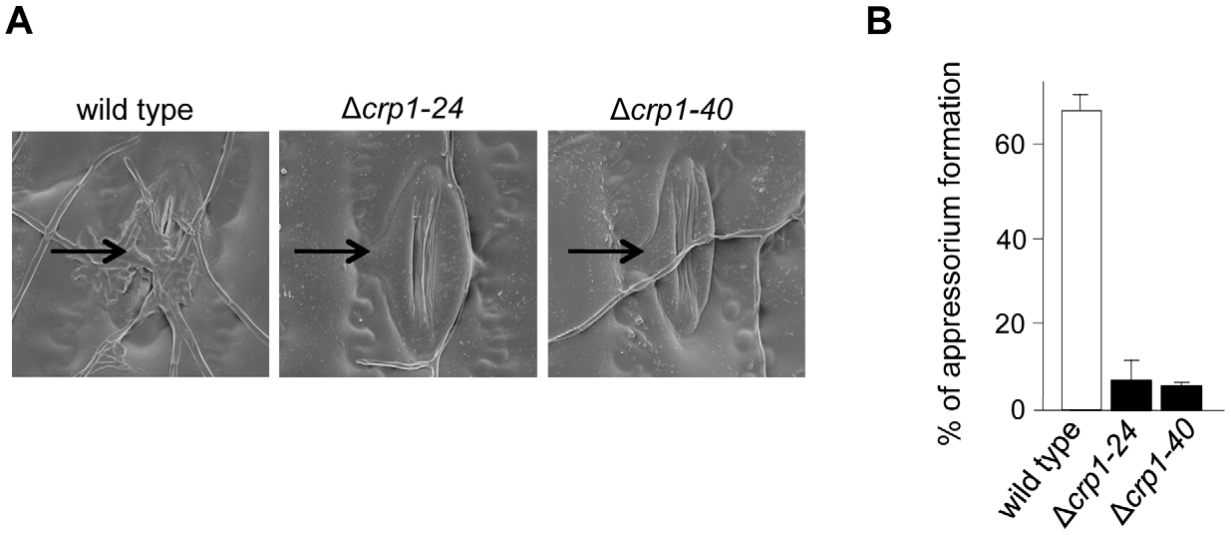
*CRP1* regulates appressorium formation and stomatal tropism in *C. zeae-maydis*. **A,** leaves inoculated with wild type, the Δ*crp1-24* mutant, and Δ*crp1-40* mutant were observed with electron microscopy. Stomata are indicated with black arrows. **B,** ratio of appressoria formed per stomate encountered for the wild type and Δ*crp1* mutants. Ratio was calculated by counting how many appressoria were formed by 100 arbitrarily selected hypha that had physically encountered a stomate.

To further dissect the role of light in the regulation of stomatal tropism and pathogenesis, we disrupted *CRP1* in *C. zeae-maydis* by homologous recombination (Figure S2A). We obtained two independent gene-disruption mutants of *CRP1* (Δ*crp1-24* and Δ*crp1-40*; Figure S2B) that were morphologically indistinguishable during growth on a wide variety of culture media (data not shown). To determine the role of *CRP1* in pathogenesis, maize plants were inoculated individually with the wild-type strain, the Δ*crp1-24* mutant, or the Δ*crp1-40* mutant. In the wild-type strain, hyphae frequently reoriented growth in the direction of stomata (Figure 5A), and nearly 70% of hyphae that encountered stomata formed appressoria (Figure 5B). However, hyphae of the Δ*crp1* mutants failed to exhibit stomatal tropism, and frequently grew around or across stomata (Figure 5A). The Δ*crp1* mutants were capable of producing appressoria over stomata, but at a nearly10-fold reduction in frequency compared to the wild-type strain (Figure 5B). Together, these results indicated that *CRP1* regulates both stomatal tropism and appressorium formation in *C. zeae-maydis*.

### *CRP1* is essential for the induction of foliar necrosis

**Figure 6:**
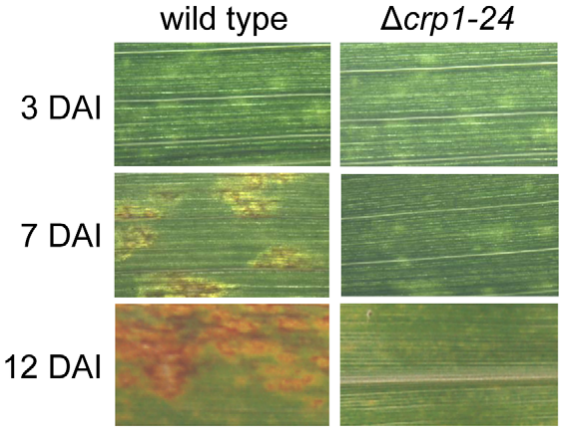
*CRP1* regulates lesion formation in *C. zeae-maydis*. Maize leaves were inoculated with a suspension of conidia (10^5^) of wild type or the Δ*crp1-24* mutant, and the inoculated maize plants were incubated in the green house for 12 days. DAI = days after inoculation.

Observations that the Δ*crp1* mutants failed to display stomatal tropism and produced low levels of appressoria prompted the question whether *CRP1* plays a broader role in colonization of host tissues and virulence. When *C. zeae-maydis* infects maize, penetration of leaves through stomata is followed by a latent phase of infection, in which the pathogen colonizes host tissue asymptomatically before switching to a necrotrophic phase of growth. The necrotrophic phase of pathogenesis results in distinctive rectangular lesions defined laterally by the minor veins of leaves. Based on the 10-fold reduction in appressorium formation in the mutant (Figure 5B), we expected to see a 10-fold or greater reduction in the number of lesions if *CRP1* regulated stomatal penetration but not colonization. However, if *CRP1* were to regulate additional components of pathogenesis, we expected either a complete failure of lesion formation or the development of non-characteristic lesions (e.g., smaller) in leaves infected by the mutant strains. On maize leaves inoculated individually with the wild type, Δ*crp1-24* mutant, or Δ*crp1-40* mutant, chlorotic flecks appeared within three days after inoculation (Figure 6). However, within seven days, leaves inoculated with the wild-type strain began to develop expanding, necrotic lesions, whereas leaves inoculated with the Δ*crp1-24* or Δ*crp1-40* mutants remained unchanged (Figure 6). After 14 days, characteristic rectangular lesions were consistently observed on leaves inoculated with the wild-type strain, whereas leaves inoculated with the Δ*crp1-24* or Δ*crp1-40* mutants failed to develop lesions in repeated inoculation attempts (Figure 6). Together, these observations indicate that *CRP1* is required for the necrotrophic phase of pathogenesis, possibly by regulating the viability of appressoria and/or the transition from latent infection to the induction of necrosis.

### Disruption of *CRP1* derepresses conidiation in constant light

**Figure 7:**
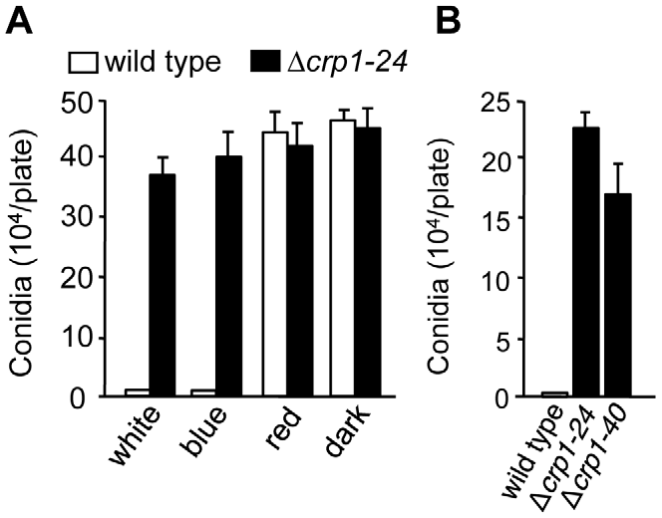
*CRP1* regulates conidiation. **A,** Conidia (10^5^) of wild type and Δ*crp1-24* were inoculated on V8-agar medium, and incubated for four days in constant white, blue, red light, or darkness. **B,** Conidia produced by the wild type and *CRP1* mutants grown on 0.2× PDA in constant white light for seven days.

To determine whether *CRP1* regulated the blue light-dependent repression of conidiation, we compared the abilities of the wild-type strain and the Δ*crp1-24* and Δ*crp1-40* mutants to produce conidia in constant light. Unlike the wild type, which grew vegetatively on V8-agar in constant while or blue light, the Δ*crp1-24* and Δ*crp1-40* mutants produced conidia at levels comparable to the wild-type strain grown in constant red light or darkness (Figure 7A), thus confirming that the repression of conidiation by blue light is mediated through *CRP1*. Surprisingly, the Δ*crp1-24* and Δ*crp1-40* mutants also produced large numbers of mature, viable conidia during growth in constant light on culture media that are typically unfavorable for conidiation (Figure 7B), thus reflecting a global de-repression of conidiation resulting from disruption of *CRP1*. In sum, these observations indicate that *CRP1* mediates the light-dependent repression of conidiation in *C. zeae-maydis* but may also regulate or respond to additional environmental inputs that influence asexual reproduction.

### Disruption of *CRP1* delays, but does not eliminate, cercosporin biosynthesis

**Figure 8:**
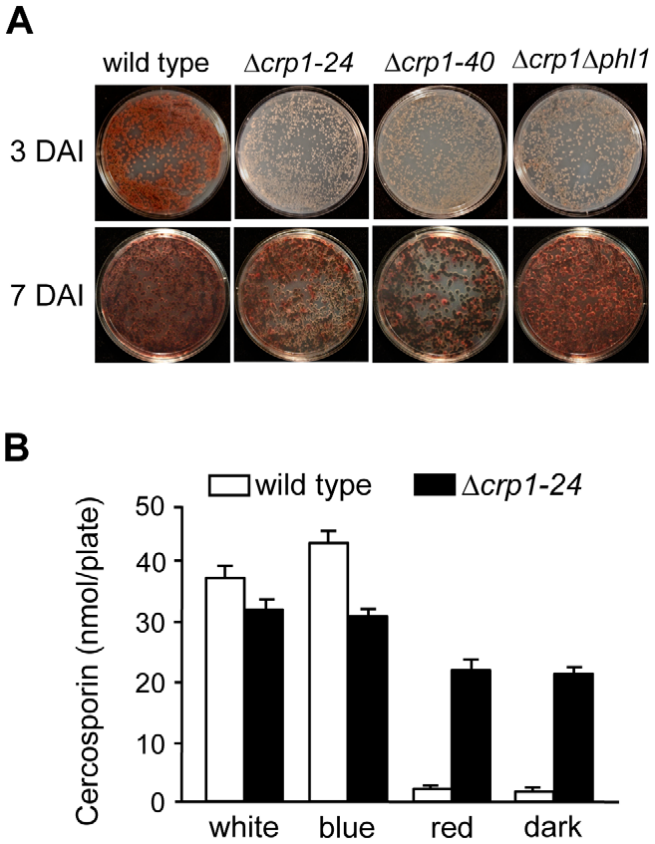
Disruption of *CRP1* affects cercosporin biosynthesis. **A,** Conidia (10^5^) were spread on 0.2× PDA, and incubated for three days and seven days in constant white light. DAI = days after inoculation. **B,** Quantification of cercosporin biosynthesis in various light conditions. Cercosporin was measured from wild type and Δ*crp1-24* cultures grown on 0.2× PDA in constant white, blue, red light and darkness for seven days.

To determine if *CRP1* regulates the induction of cercosporin biosynthesis by light, the accumulation of cercosporin in growth media was evaluated in the wild type and Δ*crp1* mutants during growth in various light conditions. When the wild type was grown on conducive media in constant white light, cercosporin accumulated as a visible pigment in the growth medium by 72 h after inoculation (Figure 8A). In comparison, the accumulation of cercosporin in culture media was delayed in the Δ*crp1-24* and Δ*crp1-40* mutants by approximately two days, but reached wild-type levels after seven days of growth (Figure 8A). Unexpectedly, in red light and darkness, the Δ*crp1-24* and Δ*crp1-40* mutants produced substantial amounts of cercosporin during growth on 0.2× PDA medium (Figure 8B), which indicated that *CRP1* is required to repress cercosporin biosynthesis in the absence of blue or white light. These findings raise two distinct possibilities: either disruption of *CRP1* leads to a constitutive, light-independent de-repression of cercosporin biosynthesis, or *C. zeae-maydis* possess another blue-light photoreceptor.

In previous work, we identified and characterized *PHL1* of *C. zeae-maydis*, which belongs to the cryptochrome/6-4 photolyase gene superfamily [18]. Functional characterization of *PHL1* indicated that the gene encodes a photolyase involved in light-dependent DNA repair, although *PHL1* and putative orthologs in other fungi appear to regulate their own expression through unknown mechanisms [18]. To explore the possibility that *PHL1* regulates aspects of blue light-dependent gene expression beyond UV-damage repair, we created a double mutant disrupted in both *CRP1* and *PHL1* (designated Δ*crp1*Δ*phl1*). When grown on 0.2× PDA medium in constant light, the accumulation of cercosporin in culture media was delayed in the Δ*crp1*Δ*phl1* mutant, as observed in the Δ*crp1-24* and Δ*crp1-40* mutants (Figure 8A). However, contrary to expectations that cercosporin production by the Δ*crp1*Δ*phl1* mutant would be less than the single knockout mutants, the Δ*crp1*Δ*phl1* mutant produced wild-type levels of cercosporin after seven days of growth, thus indicating that *PHL1* has a minimal role in the light-dependent induction of cercosporin biosynthesis.

### *CRP1* regulates photoreactivation

**Figure 9:**
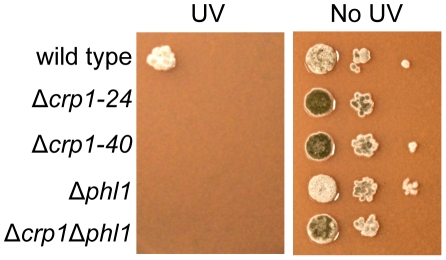
Photoreactivation of *CRP1*. Ten-fold serial dilutions of conidia (10^3^, 10^2^, and 10) from each strain were inoculated on V8-agar medium, and exposed to UV light (3 mW/cm^2^, 90 min). After UV irradiation, the plates were placed in constant light for three days.

Photoreactivation, a light-dependent mechanism through which UV-induced DNA damage is repaired, can be assessed by comparing survival after UV exposure between cultures allowed to recover in light versus darkness. In previous work, we determined that *PHL1* is required for photoreactivation, and may possess an autoregulatory mechanism for gene expression [18]. To further dissect mechanisms through which *C. zeae-maydis* perceives light, we investigated whether *CRP1* was required for photoreactivation. The wild type as well as the Δ*crp1-24*, Δ*crp1-40*, Δ*phl1, and* Δ*crp1*Δ*phl1* mutants were exposed to UV light and allowed to recover either in constant white light or darkness as previously described [18]. After three days of recovery in constant light, the wild-type strain grew robustly, but failed to grow during recovery in constant darkness; this result is consistent with previous results [18], confirming that photoreactivation occurs in the *C. zeae-maydis* wild-type strain (Figures 9, S3). However, the Δ*crp1-24* and Δ*crp1-40* mutants did not survive UV irradiation regardless of exposure to light or darkness during recovery (Figures 9, S3).

**Figure 10:**
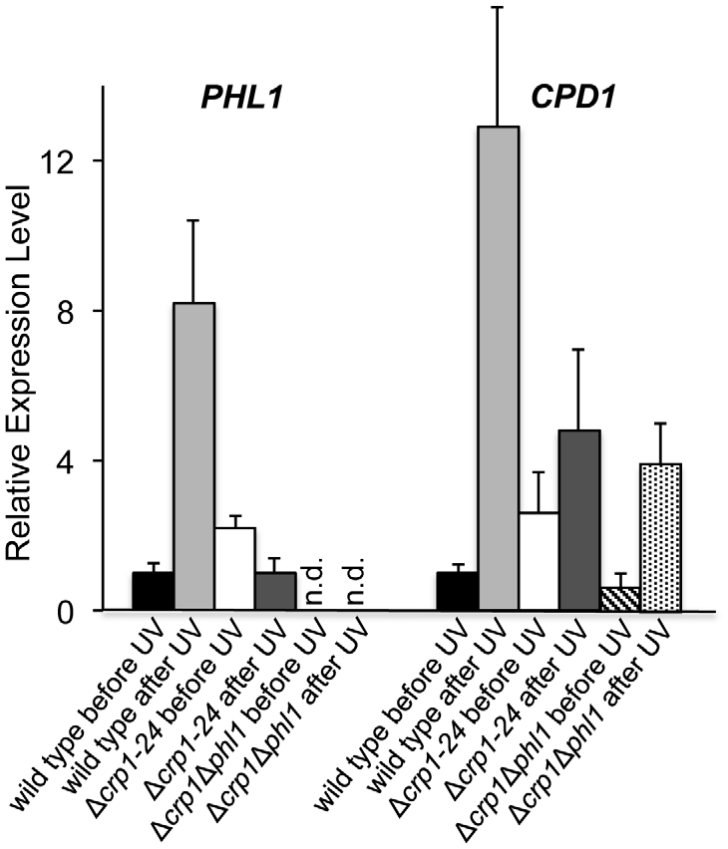
Regulation of genes involved in photoreactivation by *CRP1*. After exposure to UV light, cultures were incubated under white-light condition for 1 h to promote photoreactivation. The experiments were performed with three biological replicates, and two independent experiments produced similar results.

To clarify the mechanism through which *CRP1* regulates UV damage repair, we measured the expression of *PHL1* and *CPD1* in the wild type, Δ*crp1-24* mutant, and Δ*crp1*Δ*phl1* double mutant during photoreactivation. *PHL1* is predicted to encode a 6-4 photolyase, whereas *CPD1* is predicted to encode a cyclobutane pyrimidine dimer (CPD) photolyase in *C. zeae-maydis* [18]. After 1 h of photoreactivation, expression of *PHL1* and *CPD1* was highly induced (8 fold and 12 fold, respectively) in the wild-type strain, whereas no induction of *PHL1* was observed in the Δ*crp1-24* mutant (Figure 10). Additionally, expression levels of *CPD1* were similar in the Δ*crp1-24* mutant and the Δ*crp1*Δ*phl1* double mutant during photoreactivation (Figure 10), indicating that either *PHL1* does not directly regulate *CPD1* expression, or *CRP1* is required for any regulatory interaction between *CPD1* and *PHL1*. Although *CPD1* expression was slightly induced during photoreactivation in both the Δ*crp1-24* and Δ*crp1*Δ*phl1* mutants (Figure 9), the magnitude of the induction was comparably low in both mutants compared to the wild type. Together, these results indicated that *CRP1* regulates the induction of *PHL1* and *CPD1* during photoreactivation.

## Discussion

Infecting leaves through stomata is a critical component of pathogenesis for many fungal foliar pathogens, including *Cercospora* spp., but the process is poorly understood at the molecular level. This study presents the first molecular confirmation that stomatal tropism and infection are not random or arbitrary processes in *C. zeae-maydis*, thus validating predictions for *Cercospora* ssp. first outlined nearly a century ago. However, the precise mechanism through which *CRP1* regulates early infection events requires further elucidation. One intriguing finding in this study was that disruption of *CRP1* substantially reduced, but did not completely eliminate, the formation of appressoria over stomata; however, despite numerous, repeated foliar inoculations, we never observed the induction of foliar necrosis by the Δ*crp1* mutants. Given that infection of maize by *C. zeae-maydis* is characterized by a latent period of infection after the formation of appressoria and preceding the visible manifestation of necrotic lesions, there are at least two possible mechanisms that could explain the failure of the Δ*crp1* mutants to induce foliar necrosis. One possible explanation is that appressoria formed by the Δ*crp1* mutants have subtle morphological and/or physiological defects, and thus fail to facilitate the entry of the pathogen into the sub-stomatal cavity due to the formation of defective penetration pegs or an inability to suppress host detection. An inability to suppress host detection seems somewhat unlikely, however, considering that we never observed a distinct physiological response in the host (e.g., a hypersensitive response) induced by the Δ*crp1* mutants compared to the wild type; a visible manifestation of defense would be the anticipated result of a failure to suppress host detection. A second explanation is that the Δ*crp1* mutants may successfully penetrate leaves and enter the latent phase of infection, but fail to transition to a necrotrophic growth habit. In this scenario, the light-dependent induction of critical phytotoxins such as cercosporin and possibly other genes and/or metabolites required for the necrotrophic phase of pathogenesis would require the function of Crp1. This hypothesis is difficult to test, however, because of the interplay between *CRP1* and early infection events, and inoculation techniques that bypass the formation of appressoria for foliar entry (such as wounding) are not yet available in this pathosystem.

Although the induction of cercosporin biosynthesis by light is well established among *Cercospora* species [19], [44], [45], the mechanisms through which light is perceived and transduced to activate cercosporin biosynthetic genes have been elusive. Over thirty years ago, Lynch and Geoghehan (1979) postulated that cercosporin may function as a photopigment among the *Cercospora*, a hypothesis based on an observed overlap between wavelengths of light that strongly induced cercosporin biosynthesis in *C. beticola* [46] and the wavelength of maximum absorption (λ*_max_*) of cercosporin [45]. In the ensuing decades, many categories of chromoproteins have been discovered and characterized at the molecular level, including broadly conserved plant and fungal photoreceptors [47]. However, to date, no family of fungal proteins has been identified that could plausibly utilize cercosporin as a chromophore. Additionally, studies subsequent to the work of Lynch and Geoghehan (1979) revealed that cercosporin reacts with plasma membranes to function as a phytotoxin [19], and that *Cercospora* species possess transport proteins required to avoid self-toxicity from intracellular accumulation of cercosporin [48]. These observations strongly suggest that the primary biological function of cercosporin is a phytotoxin rather than a photoreceptor-associated chromophore. Furthermore, this conclusion is supported by our findings that at least one of the blue light-dependent morphological phenotypes of *C. zeae-maydis* (e.g., the repression of conidiation) is regulated by *CRP1* during growth on media that fully repressed cercosporin biosynthesis.

In this study, we found blue light to be the specific range of light that induces cercosporin biosynthesis, but the precise mechanism through which this occurs is not fully clear. Disruption of *CRP1* delayed the onset of cercosporin biosynthesis in blue and white light but did not fully eliminate cercosporin biosynthesis, as would be expected in a simplistic regulatory model in which *CRP1* was the sole blue-light photoreceptor responsible for transducing light as a stimulus. One possible explanation is that *C. zeae-maydis* possesses an as-yet undescribed blue-light photoreceptor that is at least partially functionally redundant with Crp1. The persistence of some blue-light responses in *N. crassa* despite the deletion of *wc-1* and *wc-2* suggests the existence of an additional blue-light photoreceptor [33]. The sequenced genomes of numerous filamentous fungi contain orthologs of plant and mammalian blue-light photoreceptors, including cryptochromes and phototropins, although none has been demonstrated to function as a blue-light photoreceptor to date. However, the postulated existence of a novel blue-light photoreceptor does not adequately explain the de-repression of cercosporin biosynthesis in red light and constant darkness in the Δ*crp1* mutants. A second possible explanation for how light regulates cercosporin biosynthesis is that *CRP1* regulates the expression and/or degradation of an inhibitor of cercosporin biosynthesis. Cercosporin biosynthesis is known to be inhibited in constant light when plates are inoculated at high spore density [49], which indicates the existence of an inhibitory feedback mechanism that can at least conditionally supersede the activation of cercosporin biosynthesis by light. In this model, the basal state for cercosporin biosynthesis in *C. zeae-maydis* could be ‘off’ due to the function of an inhibitor, which would be degraded through the function of *CRP1* in response to blue light. *CRP1* could also regulate the biosynthesis of the inhibitor in a light-dependent or –independent manner, which would explain the derepression of cercosporin biosynthesis in normally non-inducive light conditions. A more thorough molecular characterization of Crp1 and a better understanding of its interaction with signaling pathways in *C. zeae-maydis* is required to support either of these working models.

Zonate, concentric rings radiating from the site of infection are common among fungal foliar diseases and could plausibly result from periodicity underlying pathogenesis. The colonization of host tissue by hemibiotrophic fungi such as *C. zeae-maydis* is hypothesized to result from a defined succession of events, beginning with the secretion of resistance-suppressing proteins and/or secondary metabolites, followed by the growth of new hyphae behind the advancing front of secreted hydrolytic enzymes that induce necrosis, and culminating in the production of conidia after successful colonization. In the example of *C. zeae-maydis*, once the fungus penetrates the intercellular space, light would induce the production of cercosporin, a potent disruptor of host-cell membranes, and the fungus would presumably colonize new tissue and produce conidia at night. In this model, the fungus would alternative between invasive growth (daytime) and reproductive growth (nighttime) during colonization of leaves. Under field conditions, lesions caused by *C. zeae-maydis* are often scalariform in appearance; our observations indicate the laddering pattern of lesions results from bands of conidiophores interspersed by zones of vegetative growth (data not shown). Additionally, a blue light-entrained circadian rhythm underlying hyphal melanization in the soybean foliar pathogen *C. kikuchii* was recently described [50]. These observations gave rise to the hypothesis that circadian rhythms regulate components of pathogenesis or fungal development among *Cercospora* spp., including *C. zeae-maydis*, and perhaps other groups of fungal foliar pathogens.

Some 100 years after the earliest documented observations that fungi can penetrate host leaves through stomata and that light plays a role in fungal pathogenesis, this study uncovered a crucial molecular component of both phenomena. By demonstrating the requirement of a putative blue-light photoreceptor in the perception of stomata, the formation of appressoria, and the subsequent colonization of host tissue, our findings raise the possibility that fungal pathogens utilize light to synchronize key elements of pathogenesis, and also challenge assumptions that the penetration of stomata by fungi is a chance occurrence. The identification of *CRP1* provides a novel avenue through which the molecular components of light perception and pathogenesis can be explored. Additionally, our findings underscore the utility of *Cercospora* species as alternative models in which to study the molecular dynamics of light signaling and molecular clocks in fungal biology and plant-fungal interactions.

## Materials and Methods

### Strains and culture conditions

Wild-type *C. zeae-maydis* (strain SCOH1-5) was isolated from diseased maize plants collected in Scott County, Ohio in 1995. From the *C. zeae-maydis* SCOH1-5 strain, Δ*crp1-24*, Δ*crp1-40*, and Δ*crp1*Δ*phl1* were generated for this study and are described in greater detail below. All strains were maintained on V8-agar medium in constant darkness to promote conidiation; conidia were harvested with sterile water and quantified with a hemocytometer. To assay conidiation and cercosporin production in response to light, freshly harvested conidia from dark-grown cultures were plated on V8-agar or 0.2× potato dextrose agar (PDA; B&D) medium, respectively.

### Phylogenetic analysis

A data set of Crp1-like proteins was assembled from public data repositories (GenBank, DOE Joint Genome Institute, and Broad Instiute) by blastP [51]. Sequences were initially aligned with Muscle [52]. Subsequently, ambiguously aligned regions were removed using Gblocks [53]. The amino acid data set was analyzed using maximum likelihood methods under the Whelan and Goldman (WAG)+gamma+estimation of proportion of invariant sites model in RAxML v 7.0.0 [54], [55] on the available CIPRES web-portal [56]. Internal branch support was evaluated in RAxML based on 1,000 bootstrap replicates.

### Light treatments

To control the wavelengths of light available to cultures, light filters were constructed from blue (400–530 nm), green (450–600 nm), orange (540–700 nm), or red (600–700 nm) acrylic glass (American Acrylics, Skokie, IL). Spectral properties of all acrylic glass filters were determined with a Beckman DU-530 scanning spectrophotometer. For all experiments, light was provided by conventional 40 W fluorescent bulbs (GE). To control the intensity of light, cultures were positioned at varying distances from light sources or covered in layers of cheesecloth so that all cultures received a constant supply of 5 µE of light. All light measurements were taken with a LiCor integrating spectrophotometer.

### Cercosporin analysis

To extract cercosporin, cultures were grown on 0.2× PDA for four days. The plates were flooded with 5 ml of 5 N KOH, and incubated for 30 min. From each plate, 1 ml of extract was diluted 1∶4 and measured with a Beckman DU-530 spectrophotometer at a wavelength of 480 nm as previously described [18]. Experiments contained three biological replications and experiments were repeated four times with similar results.

### Nucleic acid manipulations and GUS staining

For genomic DNA extractions, fungal tissue was obtained from four-day-old cultures grown in liquid YEPD medium. Fungal genomic DNA was extracted by a CTAB protocol as described previously [57]. DNA sequences were determined by the Purdue University Genomics Center (West Lafayette, IN). PCR primers were obtained from Integrated DNA Technologies (Coralville, IA). For GUS staining, tissues were incubated in a solution containing 0.1 M NaPO_4_, 10 mM EDTA, 0.1% Triton X-100, 1 mM K_3_Fe(CN)_6_, and 2 mM X-Gluc (5-Bromo-4-chloro-3-indoxyl-beta-D-glucuronide cyclohexylammonium salt; Gold Biotechnology, St. Louis, MO) for 30 min at 37°C.

### Identification and disruption of *CRP1*

To identify *CRP1*, degenerate PCR primers CRP1degF (5′-ATHAAYTAYMGNAARGGAGG-3′) and CRP1degR (5′-CCRCARCTRTTRCANAATC-3′) were designed by aligning amino acid and nucleotide sequences of predicted genes from *Magnaporthe oryzae*, *Fusarium graminearum*, *Aspergillus clavatus*, and *Ustilago maydis*. Genomic DNA of *C. zeae-maydis* was amplified with primers CRP1degF and CRP1degR to generate a 0.9-kb product that was cloned into pGEM-T EZ (Promega) and sequenced. The remainder of *CRP1* was obtained by genome-walker PCR (Clontech) and sequencing clones containing *CRP1* identified in a cosmid library containing 8× coverage of the *C. zeae-maydis* genome (kindly provided by Dr. Won-Bo Shim, Texas A&M University). In total, we sequenced 5,925 bp of the *CRP1* locus, including the entire open reading frame of *CRP1*, 1,040-bp upstream from the putative start codon, and 1,350-bp downstream from the putative stop codon.

For functional analysis of *CRP1*, we targeted the gene for disruption via single homologous recombination in wild-type strain SCOH1-5. A 644 bp region of *CRP1* was amplified with primers Crp1KOf (5′-CCGGATCCATCCATGAAGGCG-3′) and Crp1KOr (5′-GAGGATCCTGCCAAACTGCG-3′) and cloned into vector pKS-HYG [58] to create pCRP1-KO. Then, a single-homologous disruption cassette was amplified from pCRP1-KO with primers Crp1KOf and HygR (5′-CGATCAGAAACTTCTCGACAG-3′). The PCR product was precipitated and used to transform protoplasts of *C. zeae-maydis* as previously described [59]. Hygromycin-resistant colonies were screened by PCR with primers A1 (5′-ATCTCGAGGTGTACGCATGGTGCTA-3′) and H3 (5′-CGGCAATTTCGATGATGCAGCTTG-3′) to identify two independent strains disrupted in *CRP1* (Δ*crp1-24* and Δ*crp1-40*).

Additionally, to create a Δ*crp1*Δ*phl1* strain, *CRP1* was disrupted in the Δ*phl1-1* background. The methodology was essentially the same as described above for disruption in the wild-type strain. However, because *PHL1* was disrupted with a cassette conveying resistance to hygromycin, plasmid pKS-GEN conveying resistance to geneticin was used in place of pKS-HYG [60].

### SEM microscopy

Infected leaves were collected in 24 h intervals after infection. Leaves were examined at the Purdue Life Science Microscopy Facility with a JEOL JSM-840 Scanning Electron Microscope.

### Pathogenicity assays

Maize inbred B73, which is highly susceptible to infection by *C. zeae-maydis*, was inoculated in a greenhouse when plants were approximately five weeks old. Leaves were inoculated with 1 ml of conidia suspension (10^5^ conidia/ml) by an atomizer attached to an air compressor. Inoculated plants were incubated under opaque plastic bags for five days to promote symptom development. In each experiment, at least three plants were inoculated with each strain. The pathogenicity assays were performed three times with similar results.

### UV sensitivity

From each strain, 2.5 µl of conidia suspension containing 1000, 100, or 10 conidia were spotted onto V8-agar medium. The plates were exposed to UV light (3 mW/cm^2^) for 90 min and incubated for three days in constant light or darkness. As a control, without UV treatment, plates spotted by the conidia suspension (2.5 ul) were incubated for three days in constant light or darkness. Each experiment evaluated by two plates was repeated two times with similar results.

### QRT-PCR analysis

Total RNA was extracted with Trizol reagent (Invitrogen) and purified with an RNeasy miniprep purification kit (Qiagen). For analyses of gene expression, cDNA was generated with random primers by Stratascript RT-PCR system (Stratagene). Expression of *PHL1* (forward primer 5′-AGTTCTGGGATTGCTGGACCGAAA-3′, reverse primer 5′-TCTCGCCACCTTTATGAGGCGAA-3′) and *CPD1* (forward primer 5′-CTCGAATAGAGCATCGTCGTATTCCC-3′, reverse primer 5′-TGGCATGGCGGGAGTTTTACAAG-3′) was measured by quantitative PCR. The PCR reactions were performed in an MXP-3000 real-time PCR system (Stratagene), and the reaction conditions were followed by previously described [18]. To normalize expression data, *TUB2* was amplified with primers Tub-rtf (5′-GGCTGGTGAGTGGTGCGAAA-3′) and Tub-rtr (5′-GCTCAACAGCGATCTGCGCA-3′). Expression of *PHL1* and *CPD1* was normalized to *TUB2* expression and calculated as fold differences in expression relative to expression in the wild type before exposure to UV light. The calculation was based on 2^−ΔΔCt^ method.

### GenBank accession numbers

Sequence of genes and proteins mentioned in this study can be found in the GenBank (http://www.ncbi.nlm.nih.gov/) with following accession numbers: *Cercospora zeae-maydis CRP1* (HQ646376), *C. zeae-maydis PHL1* (EU443730), *C. zeae-maydis CPD1* (EU814871), *C. zeae-maydis TUB2* (EU402967), *Neurospora crassa wc-1* (CAA63964.2), *N. crassa vvd* (AAK08514.1), *Cryptococcus neoformans BWC1* (AY882437), *Phycomyces blakesleeanus MADA* (DQ229146.1), *Aspegillus clavatus lreA* (XM001270598), *Bipolaris oryzae BLR1* (AB273633).

## Supporting Information

Figure S1 Effect of blue light on cercosporin biosynthesis of *Cercospora* spp. Pictures were taken four days after inoculation on 0.2× PDA.
(TIF)

Figure S2 Disruption of *CRP1*. **A,** Disruption of *CRP1* was accomplished by inserting a hygromycin-resistance cassette (HYG^R^) into the open reading frame of the gene via homologous recombination. **B,** Identification of strains disrupted in *CRP1* (Δ*crp1*) by PCR. A size standard (1 kb DNA ladder; Invitrogen) represented by M. Two transformants were identified with primer sets A1/H3 and A2/H5 as disrupted in *CRP1* Insertion of the HYG^R^ cassette into *CRP1*.
(TIF)

Figure S3 Photoreactivation of *CRP1* (control). Ten-fold serial dilutions of conidia (10^3^, 10^2^, and 10) from each strain were inoculated on V8-agar medium, and exposed to UV light (3 mW/cm^2^, 90 min). After UV irradiation, cultures were placed in constant darkness for three days.
(TIF)

Table S1 Effects of blue light on conidiation and cercosporin biosynthesis of *Cercospora* spp.
(DOC)
