# Supplementary figures and images for: Regulation of Stomatal Tropism and Infection by Light in Cercospora zeae-maydis: Evidence for Coordinated Host/Pathogen Responses to Photoperiod?
Source: PLoS Pathog. 2011 Jul 28;7(7):e1002113. doi: 10.1371/journal.ppat.1002113 (PMC3145785; doi:10.1371/journal.ppat.1002113)

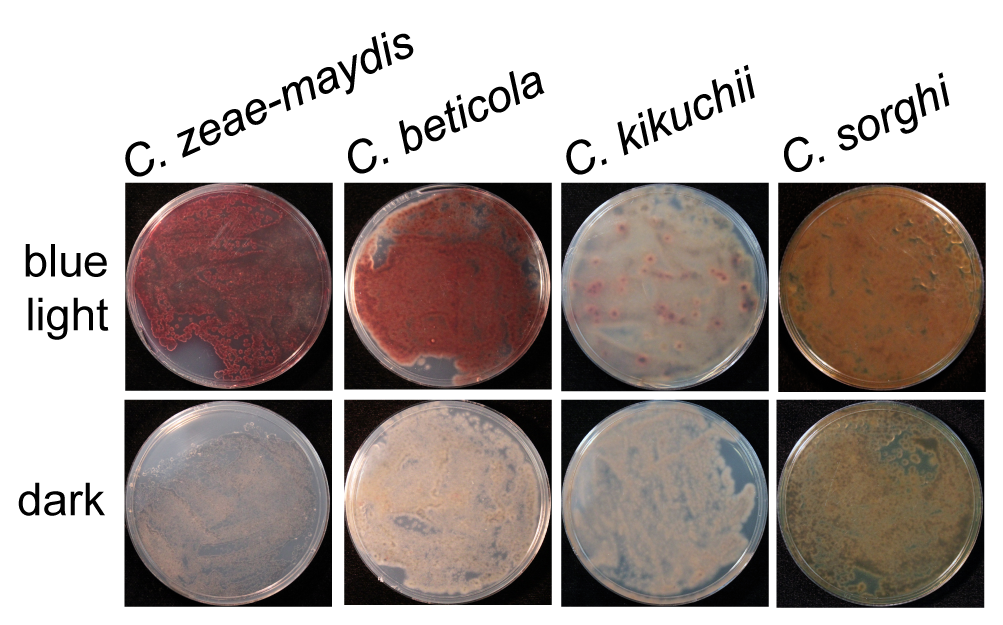

Supplement: Figure S1 — Effect of blue light on cercosporin biosynthesis of Cercospora spp. Pictures were taken four days after inoculation on 0.2× PDA. (TIF) [file ppat.1002113.s001.tif]

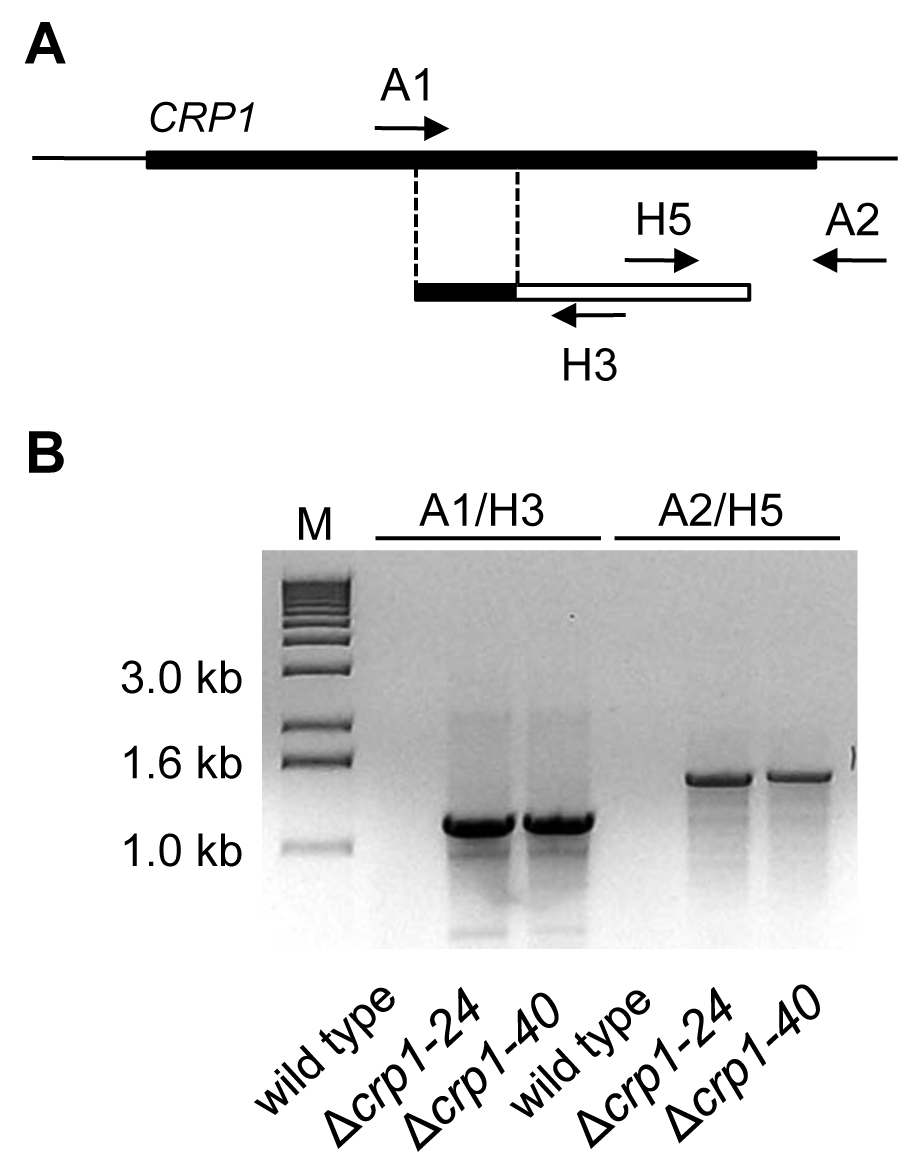

Supplement: Figure S2 — Disruption of CRP1. A, Disruption of CRP1 was accomplished by inserting a hygromycin-resistance cassette (HYGR) into the open reading frame of the gene via homologous recombination. B, Identification of strains disrupted in CRP1 (Δcrp1) by PCR. A size standard (1 kb DNA ladder; Invitrogen) represented by M. Two transformants were identified with primer sets A1/H3 and A2/H5 as disrupted in CRP1 Insertion of the HYGR cassette into CRP1. (TIF) [file ppat.1002113.s002.tif]

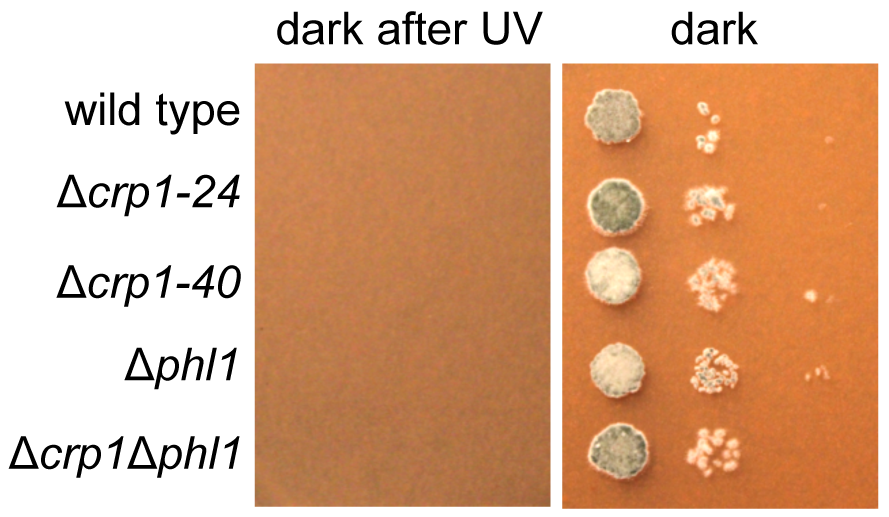

Supplement: Figure S3 — Photoreactivation of CRP1 (control). Ten-fold serial dilutions of conidia (103, 102, and 10) from each strain were inoculated on V8-agar medium, and exposed to UV light (3 mW/cm2, 90 min). After UV irradiation, cultures were placed in constant darkness for three days. (TIF) [file ppat.1002113.s003.tif]
