# Supplementary material for: Regulation of Stomatal Tropism and Infection by Light in Cercospora zeae-maydis: Evidence for Coordinated Host/Pathogen Responses to Photoperiod?
Source: PLoS Pathog. 2011 Jul 28;7(7):e1002113. doi: 10.1371/journal.ppat.1002113 (PMC3145785; doi:10.1371/journal.ppat.1002113)
Supplement: Table S1 — Effects of blue light on conidiation and cercosporin biosynthesis of Cercospora spp. (DOC) [file ppat.1002113.s004.doc]

**Table S1.** Effects of blue light on conidiation and cercosporin biosynthesis of *Cercospora* spp.

|  | Conidiation (10^5^/plate) | | Cercosporin (nmole/plate) | |
| --- | --- | --- | --- | --- |
| Species | Blue light | Darkness | Blue light | Darkness |
| *C. zeae-maydis* | 0.7 ± 0.2 | 16.3 ± 1.5 | 74.3 ± 6.7 | n.d. |
| *C. beticola* | n.d. | 12.3 ± 2.5 | 162.8 ± 13.1 | 2 ± 0.4 |
| *C. kikuchii* | 27.0 ± 9.6 | 57.0 ± 8.1 | 30.9 ± 2.3 | n.d. |
| *C. sorghi* | 8.0 ± 4.3 | 18.7 ± 5.1 | trace amount^b^ | n.d. |

^a^ Conidiation and cercosporin biosynthesis were measured in cultures grown on V8-agar and 0.2× PDA, respectively, for four days. n.d.= not detected.

^b^ Trace amount represents that the culture shows visible reddish pigment of cercosporin, but cercosporin was not detected spectrophotometrically.
